# *All-trans* retinoic acid in non-promyelocytic acute myeloid leukemia: driver lesion dependent effects on leukemic stem cells

**DOI:** 10.1080/15384101.2020.1810402

**Published:** 2020-09-08

**Authors:** Chi H. Nguyen, Alexander M. Grandits, Louise E. Purton, Heinz Sill, Rotraud Wieser

**Affiliations:** a Division of Oncology, Department of Medicine I, Medical University of Vienna, Vienna, Austria; b Comprehensive Cancer Center, Vienna, Austria; c Stem Cell Regulation Unit, St. Vincent’s Institute of Medical Research and Department of Medicine at St. Vincent’s Hospital, The University of Melbourne, Melbourne, Australia; d Division of Hematology, Medical University of Graz, Graz, Austria

**Keywords:** AML, atRA, hematopoietic stem cell, leukemia stem cell, MECOM, FLT3

## Abstract

Acute myeloid leukemia (AML) is an aggressive, often fatal hematopoietic malignancy. *All-trans* retinoic acid (atRA), one of the first molecularly targeted drugs in oncology, has greatly improved the outcome of a subtype of AML, acute promyelocytic leukemia (APL). In contrast, atRA has so far provided little therapeutic benefit in the much larger group of patients with non-APL AML. Attempts to identify genetically or molecularly defined subgroups of patients that may respond to atRA have not yielded consistent results. Since AML is a stem cell-driven disease, understanding the effectiveness of atRA may require an appreciation of its impact on AML stem cells. Recent studies reported that atRA decreased stemness of AML with an *FLT3*-ITD mutation, yet increased it in *AML1-ETO* driven or *EVI1*-overexpressing AML. This review summarizes the role of atRA in normal hematopoiesis and in AML, focusing on its impact on AML stem cells.

## Introduction

Acute myeloid leukemia (AML) is a genetically heterogeneous disease, in which a number of recurrent genetic and molecular alterations are predictive of response to therapy [1–4]. Until recently, the great majority of patients were treated with conventional chemotherapy, and the only targeted drug used in routine clinical practice was *all-trans* retinoic acid (atRA), which is highly effective in a subgroup of AML characterized by rearrangements of the retinoic acid (RA) receptor, RARA [5–7]. Even though *in vitro*, atRA promoted blast cell differentiation, originally considered its key anti-leukemic activity, also in AML without RARA rearrangements, clinical trials did not convincingly demonstrate therapeutic utility [8–15]. Since AML is a stem cell-driven disease, a small number of studies has recently addressed the impact of atRA on leukemic stem cells (LSCs) and found that it varied widely depending on the identity of the respective driver lesions [9,16,17]. Additionally, the primitivity of an LSC is likely to influence its response to atRA [17]. This review summarizes the role of atRA in normal and leukemic hematopoiesis, with a focus on its effects on LSCs. Specific consideration is also given to *EVI1* (*MECOM, PRDM3*), a gene with key roles both in HSCs [18] and LSCs [17].

## Acute myeloid leukemia

In order to sustain the life-long renewal of blood cells, hematopoiesis is organized in a hierarchical manner. The apex of this hierarchy is formed by hematopoietic stem cells (HSCs), a rare, mostly quiescent cell type that resides in a specialized niche in the bone marrow (BM) and is able to both self-renew and give rise to proliferatively active, progressively differentiating progenitor cells [19–21]. Mutations accumulating in hematopoietic stem and progenitor cells (HSPCs) over the lifetime of an individual can lead to malignant transformation [22]. One of the most aggressive hematopoietic malignancies is acute myeloid leukemia (AML), which has an annual incidence of 3–8/100,000 and a median age of onset of around 67 years [5,6]. AML is characterized by the accumulation of immature blasts at the expense of normal myeloid cells in BM and often also peripheral blood (PB), leading to anemia, bleeding, infections, and, if left untreated, death within months. By analogy to normal hematopoiesis, leukemic hematopoiesis emerges from leukemic stem cells, which reside in the hematopoietic niche of the BM and are mostly quiescent, but able to self-renew and give rise to proliferatively active progeny [23–27]. Moreover, LSCs are considered to be able to survive chemotherapy and give rise to relapse [23–27]. Even though the view that LSCs are resistant to conventional cytotoxic therapy has been challenged recently [26], it is supported by observations that high LSC frequencies, as well as the presence of stem cell expression signatures, correlate with inferior outcome in AML [4,23,24,28].

The transforming events giving rise to an LSC may take place either in an HSC or in a progenitor cell that consequently regains stem cell characteristics [23,24,26,29]. They include cytogenetic aberrations, point mutations, copy-number alterations, and epigenetic and transcriptional changes [1–4,30]. Leukemogenic mutations occur in a nonrandom order: alterations in genes coding for epigenetic regulators and chromatin remodeling factors appear prior to mutations in genes coding for transcription factors and signaling molecules [3,31–34]. Remarkably, early-type mutations were also found in phenotypically and functionally normal HSCs in some patients with AML [31–35], and even in a subset of healthy individuals [36–38]. This has led to the concept of pre-LSCs, i.e., stem cells bearing early leukemogenic driver mutations but not yet fully transformed [39]. Aberrations recurring in the malignant cells of different patients may act as drivers of leukemogenesis, represent prognostic markers, and serve as targets for rationally designed therapies [1–4,40,41].

Standard treatment for the majority of patients with AML consists of chemotherapy based on cytosine arabinoside (araC) and an anthracycline for induction. Consolidation comprises further chemotherapy, sometimes complemented by HSC transplantation [42–44]. However, 5-year survival ranges only between <5 and ~40%, depending on a variety of prognostic parameters, e.g., age, white blood cell count, and the presence of specific genetic and gene expression alterations [2,5,6]. Recently, several targeted therapeutics, including tyrosine kinase inhibitors, BCL2 inhibitors, IDH inhibitors, and antibody-drug conjugates, have been approved for the treatment of AML [41]. Notably, one of the first examples of a molecularly targeted anti-cancer drug, albeit discovered without knowledge about its mechanism of action, is *all-trans* retinoic acid (atRA). atRA has greatly improved the outcome of acute promyelocytic leukemia (APL), a subtype of AML characterized by expression of an aberrant retinoic acid receptor [45–47].

## atRA and its roles in normal hematopoiesis

atRA, the major biologically active metabolite of vitamin A, plays multiple roles during development and in the adult organism [48–50]. Conversion of vitamin A (retinol) into atRA requires two sequential oxidation steps, of which the second, irreversible one is catalyzed by members of the aldehyde dehydrogenase (ALDH) family, also known as retinaldehyde dehydrogenases (RALDHs)[51]. Conversely, atRA catabolism is initiated by cytochrome p450 (CYP) enzymes, primarily of the CYP26 subfamily [51]. atRA exerts its biological effects mainly through nuclear receptor type transcription factors composed of a retinoic acid receptor (RAR) and a retinoid X receptor (RXR) subunit. Each of these subunits has three isoforms that are encoded by paralogous genes – *RARA, RARB, RARG*, and *RXRA, RXRB, RXRG*, respectively, with additional diversification through alternative splicing [48,52,53]. The RAR/RXR heterodimer binds to specific retinoic acid response elements (RAREs) in the regulatory regions of numerous target genes, repressing their transcription in the absence of ligand and activating it in its presence [48,52,53]. RAR activation is followed by its degradation via the ubiquitin/proteasome pathway [53,54].

**Figure 1.**
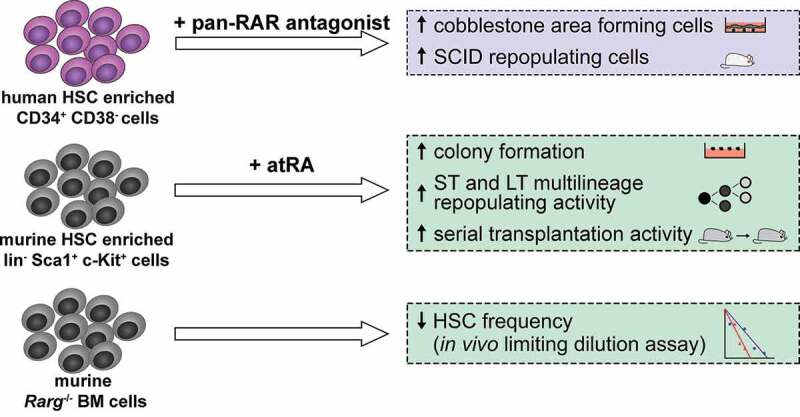
Role of *all-trans* retinoic acid (atRA) in hematopoietic stem cells (HSCs). Blue box summarizes key experiments leading to the conclusion that atRA negatively affects HSCs. Green boxes summarize key experiments leading to the conclusion that atRA positively affects HSCs. RAR, retinoic acid receptor; SCID, mice with severe combined immunodeficiency; ST, short term; LT, long term. Human cells are depicted in purple and murine cells in gray. The number of symbols in the serial transplantation assay is not meant to indicate the actual number of transplantations

atRA plays several well-established roles in hematopoiesis, among them the promotion of granulocytic differentiation of committed progenitor cells [49,55–57]. In contrast, its roles in HSCs were controversially described (Figure 1). Some reports suggested that HSCs are subject to negative regulation by atRA: microarray analysis of human HSC enriched CD34^+^ CD38^−^ cells and progenitor enriched CD34^+^ CD38^+^ cells suggested that the RA pathway was down-regulated in HSCs [58]. *In vitro* treatment with a pan-RAR antagonist increased the numbers of “cobblestone area forming cells-week 8” (CAFCW8) and of cells with the ability to repopulate severe combined immunodeficiency (SCID) mice (SCID repopulating cells, SRCs), both considered as readouts of human HSC activity. Likewise, co-culture of CD34^+^ CD38^−^ cells with stromal cells maintained their CAFCW8 activity and SRC numbers. These effects were partially counteracted by chemical or genetic inhibition of CYP26, suggesting that stromal cells contributed to HSC maintenance by inactivating RA [58]. In a related study, an RXR antagonist maintained human lineage marker negative (lin^−^) CD34^+^ CD38^−^ cells in G_0_ during culture, and substantially increased their non-obese diabetic (NOD) SCID repopulating frequency [59]. Furthermore, genetic or pharmacological inhibition of ALDH activity, and thus, presumably, RA synthesis, increased the radioprotective cell frequency and the short term (ST) repopulating potential of immunophenotypically defined, HSC enriched human and murine cell populations [60,61]. However, ALDH inhibition had no effect on the long term (LT) repopulating ability of murine HSPCs [61], indicating that its activity did not inhibit the most primitive stem cells.

In studies using murine HSPCs, *in vitro* exposure of HSC enriched lin^−^ Sca1^+^ c-Kit^+^ (LSK) cells to the physiological agent atRA enhanced their proliferation and maintained a more immature cell surface marker profile, prolonging their ability to form immature hematopoietic colonies in semisolid media [56]. Importantly, LSK cells cultured with atRA had increased ST and LT multilineage repopulating ability in a competitive repopulation assay, while the pan-RAR antagonist AGN193109 abrogated these activities [62]. The LSK cells cultured with atRA displayed increased repopulation during serial transplantation studies, which are the gold standard test for HSC self-renewal [63]. The contrasting effects of atRA on myeloid differentiation and on HSCs were attributed to the activity of different RAR isoforms. *In vitro* experiments after experimental expression of RAR isoforms, as well as competitive repopulation and *in vivo* limited dilution assays with cells from *Rara* and *Rarg* knock-out mice suggested that RARA promoted myeloid differentiation, while RARG mediated HSC maintenance by atRA [63].

Genome-wide gene expression profiling experiments taking advantage of the refined knowledge of the immunophenotypes of murine HSPCs revealed that atRA signaling was highly enriched in dormant HSCs *versus* activated HSCs and early myeloid progenitor cells [64]. *In vitro* and *in vivo* treatment with atRA enhanced HSC quiescence and serial replating and serial transplantation activity, even under HSC activating stress conditions. By contrast, maintenance of mice on a vitamin A free diet for ~4 months decreased HSC quiescence and activity [64].

Possible explanations for the partially discrepant results regarding the effects of atRA on HSCs include species effects, which may reflect real differences or technical aspects (e.g., the different surface markers used to define human and murine HSPCs, and/or the need to assess human HSC activity in potentially artifact-prone xenograft assays). Also, differences in retinoid treatment – concentration, duration, and *ex* vs. *in vivo* exposure – and between the assays used may play a role [53,65]. Remarkably, in the studies claiming an inhibitory effect of atRA on HSCs, few if any experiments employed the physiological ligand itself, but rather, conclusions were mostly based on data obtained with synthetic retinoids and inhibitors. In summary, unless murine HSCs should unexpectedly behave fundamentally different from human ones, strong evidence from experiments combining agonist and antagonist treatment with knock-out models and the most stringent stem cell assays favors the interpretation that atRA promotes the abundance and activity of HSCs.

## atRA in acute promyelocytic leukemia

Acute promyelocytic leukemia (APL) is a subtype of AML that is characterized by rearrangements of the *RARA* gene. A number of different fusion partners have been described, but about 95% of cases harbor a t(15;17)(q22;q21), which fuses the *promyelocytic leukemia* (*PML*) gene to *RARA* [45,46,66]. The resulting PML-RARA fusion protein acts in a dominant negative manner on both the PML and RARA pathways, but the activity of both fusion partners is at least partially restored by pharmacological doses of atRA [45,66]. Remarkably, the inclusion of atRA in the therapy of APL a few decades ago has transformed the prognosis of this disease from very poor to highly favorable [45,47,66]. Nevertheless, atRA monotherapy, even though able to enhance APL blast differentiation and effect complete morphological remissions, does not lead to long-term disease-free survival [45,66]. The outcome is ameliorated with liposomal delivery, which achieves higher intracellular atRA concentrations and definitive cures in a proportion of cases [66]. Current treatment regimens combine atRA with anthracyclines or the even more effective arsenic trioxide (ATO) and attain long-term survival in the vast majority of patients [7,47,67].

The molecular and biological mechanisms explaining the success of atRA-based therapies in APL were addressed only after the discovery of their clinical effectiveness. As mentioned above, PML-RARA hinders the functions of both of its fusion partners. It disrupts the formation of nuclear bodies (NBs), subcellular structures in whose genesis the tumor suppressor PML plays a key role, and which regulate multiple cellular functions including proliferation, apoptosis, and senescence [45,66]. It also interferes with RARA mediated transcription activation, which promotes normal granulocytic differentiation [45,66]. PML-RARA is unresponsive to physiological levels of atRA, but pharmacological atRA concentrations cause degradation of the fusion protein and restore NB formation, transcription of RARA target genes, and myeloid differentiation [45,66]. However, in contrast to initial assumptions, the induction of myeloid differentiation appears to be insufficient to cure APL. Thus, synthetic retinoids that were able to activate transcription by RARA and PML-RARA, but not their degradation, mediated granulocytic differentiation of APL blasts, but conferred a much smaller survival benefit than atRA in a mouse model of APL [54] (with the drawback that atRA and the synthetic retinoids were administered *via* different routes). This differential impact on survival was observed both in the originally treated mice and in secondary recipients transplanted with their BM cells, the latter being considered a readout of leukemia initiating cell (LIC) activity [54]. In contrast to the effects of synthetic retinoids, increasing doses of atRA caused increasing PML-RARA degradation in APL mice, which correlated with survival benefits both for the treated mice and for secondary recipients [68]. Together, these data indicated that restoration of RARA target gene expression and APL blast differentiation are insufficient to cure APL, but rather, PML-RARA degradation and eradication of LICs are required toward this end [45,66]. In further support of this conclusion, not only atRA, acting via the RARA-moiety, but also ATO, through the PML-moiety, caused degradation of PML-RARA. atRA and ATO cooperated both with respect to PML-RARA degradation and the survival of treated APL mice and secondary recipients [66,69]. Thus, atRA at high doses and/or in combination with ATO is able to reduce LIC activity in APL, and this reduction correlates with the clinical effectiveness of a specific therapeutic regimen.

Even though not undisputed, the presence of specific additional molecular and genetic lesions, in particular, the kinase activating *FLT3* internal tandem duplication (ITD) may modulate the response of APL patients to atRA-based therapy [47,70]. In mice bearing a *PML-RARA* transgene, the additional presence of an *FLT3*-ITD knock-in allele reduced the effects of atRA on PML-RARA degradation, NB re-formation, granulocytic differentiation, *in vivo* blast clearance, and on the delay of disease onset in secondary recipients [71]. In contrast, but in agreement with the known clinical effectiveness of combined treatment with atRA and ATO in patients with *FLT3*-ITD APL, this combination promoted all of the above parameters irrespective of the presence of the *FLT3* mutation [71]. Somewhat at odds with the atRA resistance conferred by the *FLT3*-ITD in the mouse model, an exome sequencing study on matched diagnosis-relapse samples from patients with APL that had been treated with atRA plus chemotherapy showed that *FLT3* mutations present at diagnosis were consistently lost at relapse [72].

## atRA in non-APL AML: clinical trials

The tremendous success of atRA in APL, together with laboratory observations that atRA promoted differentiation and chemotherapy sensitivity of non-APL AML blasts [8–14,73,74], inspired numerous trials addressing the clinical benefit of adding atRA to chemotherapy also in non-APL AML. In a phase III trial that included 242 elderly (>60 years) patients with AML, atRA, started 2 days after cytotoxic therapy, was associated with a higher response rate, and with longer event free (EFS) and overall survival (OS) as an independent parameter [75]. Later, this trial was re-analyzed for the possible predictive power of some prognostically relevant recurrent mutations in AML, namely, the *NPM1, FLT3*-ITD, *FLT3* tyrosine kinase domain (TKD), *MLL* partial tandem duplication (PTD), and *CEBPA* mutations. This analysis suggested that the beneficial effects of atRA were restricted to the (relatively small) subgroup of patients that had a mutated *NPM1* gene but no *FLT3*-ITD [76]. In another trial, 83 AML patients >60 years received standard chemotherapy with or without atRA, and the group of patients with below-median expression of the transcriptional co-factor *MN1* experienced improved EFS and OS with atRA [8]. In contrast, in a study in which 1075 patients <60 years with non-APL AML or high-risk myelodysplastic syndrome (MDS) were randomized to receive atRA or not, atRA had no effect on response rate or survival [77]. This was true for the entire cohort, for patients with cytogenetically normal AML, and for each of the subgroups defined by *FLT3*-ITD, *NPM1*, or *CEBPA* mutations, or by *MN1* expression [77]. Also, in a randomized study of atRA in 1100 adults <60 years with AML, atRA, started on treatment day 6, did not reveal any consistent benefit in either the entire cohort or in the subgroup of patients with *NPM1* mutations [78]. A recent meta-analysis summarized eight trials comparing chemotherapy plus atRA with chemotherapy alone in a total of almost 4000 adult patients with AML, and concluded that there was no evidence for an effect of atRA on either the risk of adverse events, or on response rate, disease-free survival (DFS), or OS [15]. It should be pointed out that all discussed trials differed in numerous parameters, including patient age, the identities of the cytotoxic drugs used in conjunction with atRA, and treatment schedules, thereby precluding direct comparisons. In contrast to the so far mostly disappointing results regarding the combination of atRA with conventional chemotherapy, two recent studies suggested that atRA may be beneficial when combined with hypomethylating agents in elderly patients ineligible for induction chemotherapy [79,80]. It is assumed that hypomethylating treatment primes myeloid differentiation genes for transcription activation by RARs [81]. These recent publications, as well as the registration of at least eight currently recruiting clinical trials (clinicaltrials.gov, accessed on May 13, 2020), indicate the strong ongoing interest in exploring the activity of atRA in non-APL AML.

## atRA in non-APL AML: preclinical studies

**Figure 2.**
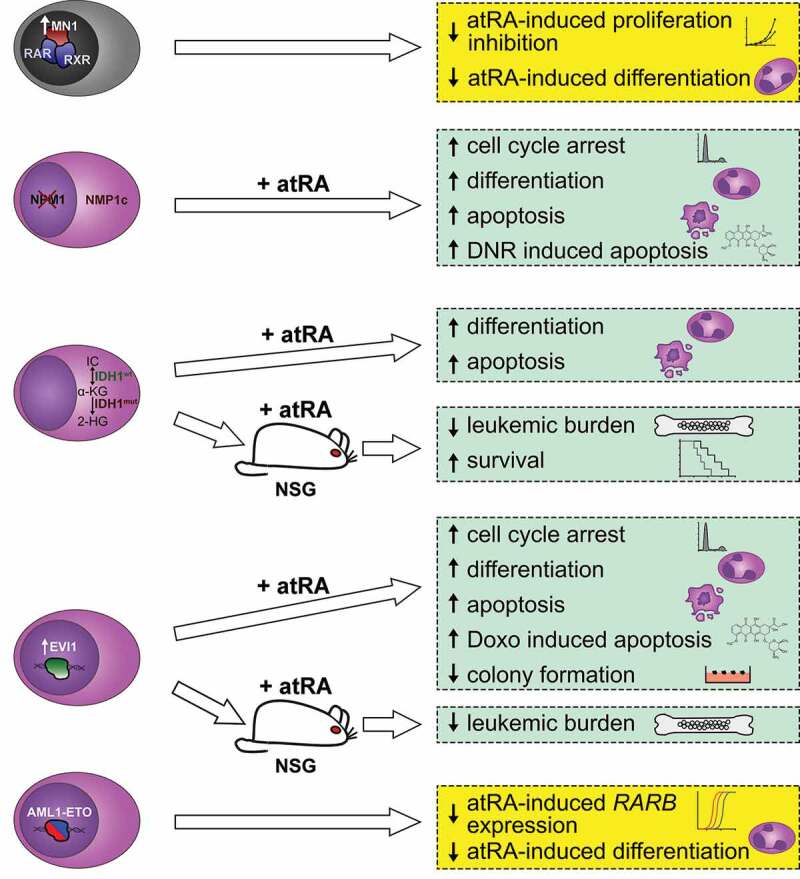
Effects of *all-trans* retinoic acid (atRA) on leukemic blasts of genetically or molecularly defined subgroups of AML. Yellow boxes summarize inhibition of anti-leukemic effects of atRA (note, however, that primary *AML1-ETO* positive blasts were found to be atRA sensitive in an independent study; see main text). Green boxes summarize anti-leukemic effects of atRA. Human cells are depicted in purple and murine cells in gray. IC, isocitrate; α-KG, α-ketoglutarate; 2-HG, 2-hydroxyglutarate; NSG, non-obese diabetic severe combined immunodeficiency IL2Rg^null^ mice; DNR, daunorubicin; Doxo, doxorubicin

Complementing the clinical trials, numerous laboratory studies have tried to identify subgroups of AML potentially benefitting from atRA, as well as agents that may sensitize resistant AML cells to retinoids (Figure 2). *MN1* encodes a transcription co-factor of the RAR/RXR complex, and its elevated expression was associated with atRA resistance in one of the clinical trials [8]. Accompanying laboratory work showed that overexpression of *MN1* greatly decreased the sensitivity of a preleukemic BM cell line toward the proliferation inhibiting and differentiation promoting effects of atRA [8]. Inducible expression of *MN1* in a human myeloid cell line enhanced or repressed the effects of atRA in a gene-specific manner [82].

Mutations in *NPM1* represent the most frequent recurrent mutation in AML, and lead to a predominantly cytoplasmic localization of the encoded chaperone protein, which usually shuttles between nucleus, nucleolus, and cytoplasm. Building on the reported clinical association between *NPM1* mutations and atRA responsiveness [76], the effects of atRA and ATO on AML cell lines and primary AML samples with and without *NPM1* mutations were investigated. Both agents, and even more strongly their combination, caused proteasome-dependent down-regulation of mutated, but not wild type, NPM1, leading to re-localization of wild type NPM1 (produced from the second allele) to the nucleus [11,12]. This was accompanied by a higher propensity of *NPM1*-mutated AML cells to respond to atRA- and/or ATO-mediated cell cycle arrest, differentiation, and apoptosis [11,12]. Furthermore, pretreatment of an *NPM1*-mutated cell line with atRA and/or ATO sensitized it to daunorubicin [11]. Compassionate use of atRA and arsenic in five elderly patients with *NPM1-*mutated AML that were deemed unfit for chemotherapy led to transient anti-leukemic effects in three of them [12].

Pertinent clinical trials had not been analyzed for an effect of mutations in the genes encoding the tricarboxylic acid cycle enzymes IDH1 and IDH2 on atRA responsiveness, but laboratory experiments suggested a possible relation [13]. *IDH1*-mutated cell lines and primary samples were more sensitive to atRA induced differentiation and apoptosis than their *IDH1* wild type counterparts, and this was counteracted by an inhibitor of mutated IDH1. In a mouse xenograft model, atRA reduced leukemic burden and increased survival in an *IDH1*-mutation-specific manner. Most provokingly, pre-treatment with a cell permeable form of 2-hydroxyglutarate, product of the neomorphic IDH1 variant and usually considered an oncometabolite, sensitized AML cell lines with wild-type *IDH1* to atRA induced differentiation [13].

The *ecotropic viral integration site 1* (*EVI1*) gene encodes a transcription factor that fulfills essential functions in HSCs, but is down-regulated during normal hematopoietic differentiation [18,83–85]. Its overexpression, observed in approximately 10% of patients with AML, is associated with a particularly poor prognosis [86–88]. Perhaps counter-intuitively, *EVI1* expression was up-regulated by atRA in cell lines and in primary AML cells [14,89–91], due to both mRNA stabilization and transcriptional up-regulation through a canonical RARE [90,91]. EVI1 counteracted its own induction by atRA but enhanced that of the *RARB* gene in luciferase reporter assays [91]. Genome-wide gene expression analysis of human myeloid cell lines with or without experimental *EVI1* expression showed that EVI1 enhanced the transcriptional responses to atRA of a number of genes [10]. Accordingly, *EVI1* also augmented atRA induced cell cycle arrest, differentiation, and apoptosis in these cell lines [10]. In primary AML blasts, atRA enhanced differentiation and apoptosis, and decreased clonogenic activity and engraftment in immunodeficient mice predominantly of EVI1^high^, but not EVI1^low^, samples [14]. Preincubation with atRA also increased the doxorubicin sensitivity of two EVI1^high^ AML samples [14]. This led to the suggestion that patients with EVI1^high^ AML may specifically benefit from atRA containing therapy, but this assumption was not tested in the relevant clinical trials, and not affirmed by studies on LSCs (see below). However, as another interesting parallel to the situation with APL and with *NPM1*-mutated AML, ATO targeted the EVI1 protein for degradation via the ubiquitin-proteasome pathway [92]. Moreover, *EVI1* overexpression appeared to confer sensitivity to ATO in murine myeloid cells and in a clinical trial including 28 patients with MDS [93].

In contrast to these studies, which identified lesions or gene expression states potentially sensitizing AML cells to atRA, the AML associated transcription factor fusion protein AML1-ETO was reported to confer atRA resistance [94]. In human myeloid cell lines, AML1-ETO recruited transcription corepressors to the RARE of the *RARB* gene, mediated increased DNA methylation and decreased histone acetylation, and prevented induction of *RARB* by atRA. An shRNA against *AML1-ETO* or treatment with 5-azacytidine reduced methylation of the *RARB* regulatory region, and restored the induction of *RARB* and differentiation in response to atRA. *RARB* methylation was also found in the majority of primary samples from patients with AML M2 or M4, morphological subtypes of AML often associated with the expression of AML1-ETO or functionally related fusion proteins [94]. In an independent study, however, *AML1-ETO* expressing primary AML blasts were found to be atRA sensitive [95].

Some authors raised the question whether the RAR pathway is functional in AML at all, since a number of genes that are mutated or misexpressed in this disease have a negative impact on it [96]. Along these lines, some reports intended to identify agents that could sensitize AML cells to atRA. As already mentioned, a cell permeable form of 2-hydroxyglutarate as well as 5-azacytidine augmented the effects of atRA on the differentiation of certain AML cell lines [13,94]. Experiments with cell lines, xenografts, and primary AML samples indicated that inhibition of the SUMO pathway may sensitize AML cells to atRA [97]. Similarly, the endoplasmatic reticulum-stress inducing drug tunicamycin was reported to cooperate with atRA and ATO to inhibit the clonogenic capacity and to promote death of human AML cell lines and primary AML cells, particularly those with a *FLT3*-ITD [98]. Another set of *in vitro* experiments led to the suggestion that inhibition of the histone acetyltransferase GCN5 and/or the lysine demethylase LSD1 augmented the anti-leukemic activities of atRA [99].

In contrast to the above described efforts, some authors have questioned the availability of atRA in the BM niche. In one study, hematopoietic cells were proposed to reside in a retinoid deplete environment in the BM based on the limited activity of a synthetic reporter gene [100]. *In vitro* experiments with AML cells suggested a role of BM stroma in degrading atRA: human AML cell lines with different driver lesions (*PML-RARA, AML1-ETO, NPM1* mutations), as well as primary samples expressing *AML1-ETO* or related fusion genes, responded to atRA by differentiation and/or loss of clonogenic activity. The activity of atRA was abrogated by co-culture with BM stromal cells, and restored by incubation with a CYP26 inhibitor [95]. Accordingly, the half-life of atRA was reduced ~3-fold by stromal co-culture, but restored by CYP26 inhibition [95]. To overcome the inhibitory effects of the BM niche, the use of synthetic, CYP26-resistant retinoids was proposed. Since atRA mediated differentiation of leukemic cells via RARA, but caused feedback-induction of stromal *CYP26B1* mostly via RARG, RARA-selectivity was considered an additional advantage [101]. IRX195183 fulfilled both requirements, and was able to effect differentiation of AML cell lines even in stromal co-culture. Moreover, it controlled disease burden more effectively than atRA in an *NPM1*-mutated AML xenograft model [101]. The RARA-selective, CYP26-resistant retinoid tamibarotene is currently being tested in a phase 2 trial including patients with AML and MDS (NCT02807558) [101].

The above-described studies are mostly based on the concept of “differentiation therapy”, i.e., the assumption that promoting the differentiation of leukemic blasts will effect, or at least support, cure of the disease. However, AML is a stem cell-driven disease and therefore can be cured only by eradication of the (small) LSC population [25]. Consequently, understanding the effects of atRA in AML requires an appreciation of its impact on AML stem cells.

## Effects of atRA on AML stem cells

Both normal and cancer stem cells from a variety of tissues are characterized by high ALDH activity [102]. The human ALDH gene family contains 19 members, whose functions include the synthesis of retinoids and the metabolism of reactive aldehydes from both endogenous and exogenous sources [102,103]. ALDHs contribute to cellular resistance against a variety of anti-cancer drugs, including daunorubicin, and protect normal and cancer cells from reactive oxygen species (ROS) produced in the context of chemo- and radiotherapy [102,103]. Cellular atRA down-regulates ALDH activity as part of a negative feedback mechanism, and suppression of ALDH by exogenous atRA or synthetic retinoids sensitizes cancer cells to chemotherapeutic drugs [102]. In AML, ALDH activity displayed a complex pattern: approximately 23% of patients had a higher proportion of ALDH^+^ cells (identified by Aldefluor staining) than normal controls (median, 1.9%; ALDH-numerous AML), while the rest had substantially lower proportions of ALDH^+^ cells (ALDH-rare AML) [104]. The distribution of AML-specific aberrations between ALDH^+^ and ALDH^−^ cells, gene expression profiling, and xenotransplantation experiments suggested that ALDH-numerous AML contained a higher number of LSCs and these were present among ALDH^+^ cells [104,105]. By contrast, in ALDH-rare AML, ALDH^+^ cells were enriched for normal HSCs [104]. In ALDH-numerous AML, ALDH^+^ cells were more resistant to araC than ALDH^−^ cells [104]. Consistent with independent data showing that high ALDH levels were associated with poorer outcome in AML [103], patients with ALDH-numerous AML had worse DFS and OS [104].

**Figure 3.**
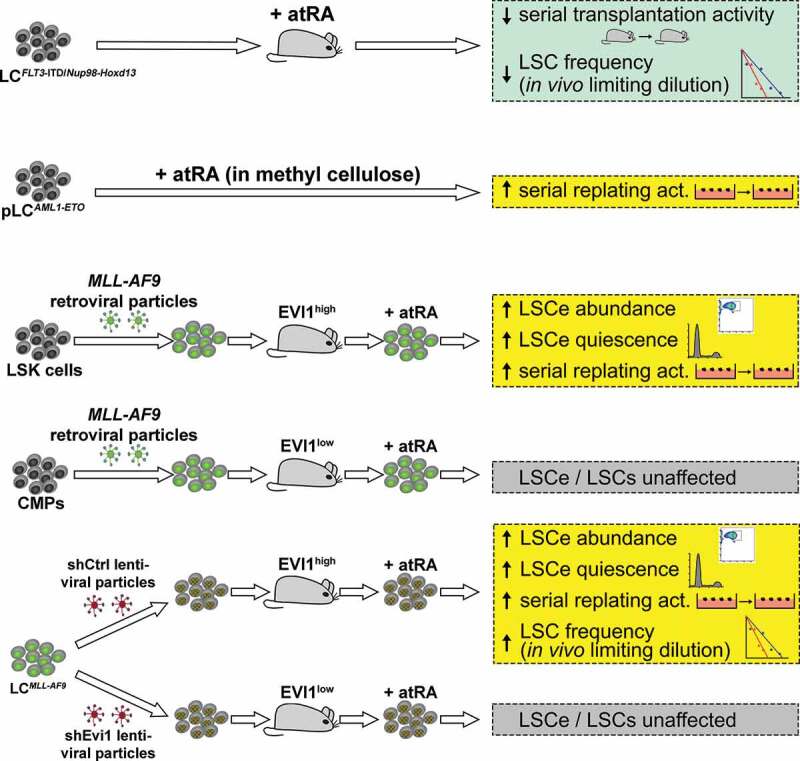
Effects of *all-trans* retinoic acid (atRA) on AML stem cells. Green box summarizes anti-leukemic effects of atRA; yellow boxes summarize pro-leukemic effects of atRA; gray boxes summarize absence of an effect of atRA. LC, leukemic cells; pLC, preleukemic cells; LSK cells, lin^−^ Sca1^+^ c-Kit^+^ cells (HSC enriched); CMPs, common myeloid progenitor cells; LSCs, leukemic stem cells; LSCe, LSC enriched cells; act., activity. Numbers of symbols in serial replating or transplantation assays are not meant to indicate the actual numbers of repetitions

While these studies can be interpreted as indirect evidence for a role of atRA in the LSCs of a subset of AML, a small number of studies has directly addressed the impact of atRA on AML stem cells, with heterogeneous results (Figure 3). Ma *et al*. investigated the effects of atRA and its interaction with the tyrosine kinase inhibitor sorafenib in AML with *FLT3*-ITD mutations. atRA enhanced the anti-leukemic effects of sorafenib in AML cell lines, primary samples, and xenografts with an *FLT3*-ITD [16]. In a congenic AML mouse model based on co-expression of an *FLT3*-ITD allele (which alone is insufficient to cause AML) with a *Nup98-Hoxd13* fusion gene, *in vivo* treatment with atRA delayed disease onset, and enhanced corresponding effects of sorafenib, in secondary recipients. An *in vivo* limiting dilution assay with cells from the treated mice revealed LSC frequencies of 1/80, 1/1,700, 1/38,000, and <1/10^6^ for vehicle, atRA, sorafenib, and atRA + sorafenib treated mice, respectively [16]. In summary, atRA not only enhanced the anti-leukemic effects of sorafenib on AML blasts with an *FLT3*-ITD mutation, but also reduced the frequency of LSCs both by itself and together with sorafenib. The ability of atRA to reduce LSC activity in *Flt3*-ITD driven AML was confirmed in an independent mouse model (Nguyen *et al*., submitted).

While atRA inhibited LSC activity in *FLT3*-ITD bearing non-APL AML, the opposite was reported for AML expressing the *AML1-ETO* fusion or overexpressing *EVI1*. atRA strongly increased the serial replating ability of *AML1-ETO* expressing murine BM cells, and resulted in larger, more immature colonies [9]. Interestingly, the effect of a RARA agonist was opposite to that of atRA, while a RARG agonist was ineffective on its own but together with the RARA agonist mimicked the effects of atRA [9]. In a congenic mouse model of *AML1-ETO*-driven AML, the RARA agonist did not prolong survival, but effected a transient decrease in leukemic burden and a persistent increase in myeloid differentiation [9].

Building on our own previous observations of a functional cooperation between *EVI1* and atRA in malignant myeloid cells [10], we asked whether similar interactions would also exist in LSCs. To this end, a mouse model of *MLL-AF9* driven AML was used. *MLL*-rearrangements are associated with *EVI1* overexpression in human AML [88,106,107]. In mice, MLL fusion proteins were able to transform both HSC enriched LSK cells and progenitor cells, and enhanced *Evi1* expression by direct promoter binding – remarkably only in LSK-, but not progenitor-, derived AML [17,106,108–111]. This suggested that the presence or absence of *EVI1* overexpression, each observed in about half of the patients, reflects the cell type in which the transforming event occurred also in human *MLL* rearranged AML [106–109,111]. Irrespective of the cell of origin, leukemic cells with the immunophenotype of granulocyte macrophage progenitors (GMPs) are strongly enriched for LSCs in *MLL-AF9* driven murine AML [109]. They were originally termed leukemic GMPs [109], but are referred to as LSC enriched cells (LSCe) by us [17].

*Ex vivo* treatment of leukemic cells (LCs) from BM of mice with LSK derived, *MLL-AF9* driven AML (LC^LSK_MLL-AF9^; EVI1^high^) with atRA augmented the abundance and quiescence of LSCe, and the activity of LSCs as determined by serial replating and *in vivo* limiting dilution assays [17]. In contrast, no such response was observed with EVI1^low^ LC^CMP_MLL-AF9^, i.e., LCs from mice that developed AML after transplantation with *MLL-AF9* transduced common myeloid progenitors (CMPs) [17]. To investigate a possible role of *Evi1* in the differential atRA responsiveness of LSCs from LSK- and CMP-derived AML, LC^LSK_MLL-AF9^ were transduced with shRNAs against *Evi1* or with a control shRNA, and transplanted into congenic recipient mice. Experiments with LCs from these mice showed that knock-down of *Evi1 per se* reduced LSCe/LSC abundance, quiescence, and activity (the first demonstration of a key role of *Evi1* in AML LSCs), and additionally abolished the stemness promoting effects of atRA [17]. *Evi1* also strongly augmented transcriptional responses of LSCe to atRA: its knock-down reduced the number of atRA-regulated genes to less than one-half the number found in control cells. Pharmacological and genetic inhibition experiments established *Notch4*, one of the joint targets of EVI1 and atRA, as a relevant mediator of their effects [17]. *Ex vivo* exposure of BM LCs to a pan-RAR antagonist affected LSCe abundance and quiescence in a manner opposite to that of atRA. Notably, *in vivo* antagonist treatment significantly prolonged survival of initially treated and secondary recipient mice, and decreased the abundance, quiescence, and activity of LSCe/LSCs. Finally, atRA increased the quiescence of human AML cell lines retaining some stem cell characteristics in an *EVI1*-dependent manner, and enhanced clonogenicity and LSCe quiescence of primary *EVI1* overexpressing, but not *EVI1*-negative, AML samples [17].

Together, these data show that atRA augmented leukemic stemness in AML resulting from HSC, but not progenitor cell, transformation. These differences could be largely explained by differential expression of the stem cell gene *Evi1*, which *per se* enhanced leukemic stemness, and additionally facilitated the stemness promoting activity of atRA, in AML.

## Summary and conclusions

The effects of atRA in the context of AML are multiple and complex. One of its earliest known and best described consequences is the promotion of myeloid differentiation of leukemic blasts, which forms the basis of most studies aiming to identify atRA-susceptible subgroups of patients, and/or substances able to sensitize AML cells to atRA [8,10–14,97-99]. atRA also augmented the susceptibility of AML cells to chemotherapeutic drugs [11,14,73,74], possibly due to its inhibitory effect on ALDHs, an enzyme family with key roles both in retinoid metabolism and drug resistance [51,102,103]. Recent studies reported that atRA regulated the abundance, properties, and activity of AML stem cells [9,16,17]. Interestingly, the nature of these effects varied widely in a manner related to the identity of the genetic driver lesions, and/or the transformed cell type. In mouse models in which AML was driven by an *FLT3*-ITD in combination with a second non-APL AML typical aberration, atRA negatively affected LSC activity [16] (and Nguyen *et al*., submitted). However, on the background of the APL-typical *PML-RARA* fusion, the *FLT3*-ITD counteracted the inhibitory effect of atRA on LIC activity [71]. In an *AML1-ETO*-driven mouse model, atRA even promoted LSC properties [9]. atRA also augmented LSC abundance, quiescence, and activity in an *MLL-AF9* driven murine AML model in a manner dependent on transformed cell type (LSK cells *vs*. CMPs) and *Evi1* expression [17]. Together, these studies highlight substantial molecularly and genetically determined heterogeneity of the effects of atRA on AML LSCs. At least partially related to this, the cell of origin also may play a role in the atRA response of LSCs: in AML arising from transformed HSCs – a cell type whose self-renewal is promoted by atRA [56,62,63] – leukemic stemness may be likewise promoted by atRA. In contrast, in AML originating from transformed myeloid progenitors, which respond to atRA by growth arrest and differentiation [49,55–57], atRA may be inert or even inhibit LSC/LIC activity.

Perhaps less surprisingly on the background of such complexity, attempts to identify atRA-responsive subgroups of patients with non-APL AML have so far not yielded conclusive results [8,15,76–78]. And even though the rationale that the effects of atRA on LSCs may explain its clinical effectiveness appears compelling, translation of corresponding laboratory data does not appear straightforward: the impact of *EVI1* overexpression on patients’ responses to atRA has not been investigated so far, and the inhibitory effect of atRA alone or in combination with sorafenib on LSCs from *FLT3*-ITD driven murine AML [16] does not correspond to clinical activity of atRA in *FLT3*-ITD positive patients [76,77]. Whether this can be explained by the fact that atRA was combined with chemotherapy rather than sorafenib in the clinical trials remains to be determined.

To be able to address the multifactorial effects of leukemia-associated molecular and genetic lesions on patients’ responses to atRA that are suggested by numerous laboratory studies, identification of atRA sensitive AML subpopulations will require large clinical trials accompanied by extensive molecular characterization. The resulting data need to be evaluated not only for the impact on atRA responsiveness of previously known prognostic parameters, but of all recurrent events and their interactions using advanced statistical tools. Such studies have the potential to reveal which patient subgroups respond to atRA or other RAR agonists, and which subgroups may even suffer a disadvantage from atRA but might benefit from RAR antagonists. The latter, certainly unorthodox possibility was suggested by our own recent work [17], and an earlier study pointed in a similar direction[9]. Given the multiple functions of atRA also in the adult organism [48–50], interfering with its pathway could be associated with substantial side effects. Nevertheless, RAR antagonists are being explored as treatments for diverse ailments, including malignancies and diseases of hematopoietic cells [17,112–119]. However, in view of the role of atRA in normal HSCs [56,62–64], the extent of a possible therapeutic window in the context of hematological malignancies requires specific consideration.

In addition to the decision between agonists and antagonists, degradation (CYP26) resistance and receptor isoform specificity are potentially relevant to the choice of retinoids with optimal anti-leukemic activity [9,101]. RARA specific agonists may be advantageous with respect to the induction of blast differentiation [9,101]. On the other hand, induction of stromal *CYP26B1* and promotion of HSC maintenance and activity by atRA was mostly ascribed to the action of RARG [63,101]. Which RAR isoforms mediate the activity of atRA toward LSCs has been investigated only in the context of *AML1-ETO*, with complex results: only the combination of a RARA and a RARG agonist mimicked the LSC-promoting effects of atRA [9]. Thus, the possible superiority of receptor isoform-specific retinoids in the context of the eradication of AML LSCs requires additional thorough assessment.

Beyond the choice of the retinoids themselves, the identity of the agents used in combination with them is likely to influence their effects. All clinical studies that investigated the effects of atRA in non-APL AML to date used different protocols[15], which may contribute to the inconsistency of their results. Possibly the most promising effects were obtained when atRA was combined with hypomethylating agents [15,79,80]. The relative timing, as well as the duration of the administration of the different drugs are also likely to be important: addition of atRA prior to or together with chemotherapy may optimize the sensitization to cytotoxic drugs, while maximal effects of retinoids on LSC elimination may be achieved when using them as maintenance therapy.

The interest in retinoids for the treatment of non-APL AML is ongoing, as evidenced by numerous recent publications and several currently recruiting clinical trials (clinicaltrials.gov). Even though the effects of atRA in AML are obviously complex, this research has important potential to lead to improved therapies for additional subgroups of patients with AML.
